# Complex Spectroscopy Studies of Nifedipine Photodegradation

**DOI:** 10.3390/pharmaceutics15112613

**Published:** 2023-11-10

**Authors:** Mirela Paraschiv, Monica Daescu, Cristina Bartha, Bogdan Chiricuta, Mihaela Baibarac

**Affiliations:** 1 National Institute of Materials Physics, 077125 Bucharest, Romania; mirela.cristea@infim.ro (M.P.); monica.daescu@infim.ro (M.D.); cristina.bartha@infim.ro (C.B.); 2 Faculty of Physics, University Bucharest, 077125 Bucharest, Romania; 3 SC Apel Laser SRL, 077135 Mogosoaia, Romania; bogdan.chiricuta@apellaser.ro

**Keywords:** nifedipine, drug photodegradation, photoluminescence, Raman scattering, IR spectroscopy, UV-VIS spectroscopy, mass spectrometry

## Abstract

The aim of this work is to highlight the influence of UV light on the hydrolysis reaction of nifedipine (NIF) in the presence of alkaline solutions. In this context, the photodegradation of NIF in the absence of alkaline solutions caused (a) a change in the ratio between the absorbances of three bands in the UV-VIS spectra localized at 224–240 nm, 272–276 nm and 310–340 nm, assigned to the electronic transitions of -COOCH_3_ groups, -NO_2_ groups and a heterocycle with six atoms; (b) a red-shift of the photoluminescence (PL) band from 458 nm to 477 nm, simultaneous with an increase in its intensity; (c) a decrease in the ratio of the Raman line intensities, which peaked at 1224 cm^−1^ and 1649 cm^−1^, associated with the vibrational modes of -C-C-O in the ester group and C=C stretching; and (d) a decrease in the ratio between the absorbances of the IR bands, which peaked at 1493 cm^−1^ and 1223 cm^−1^, associated with the vibrational modes of the -NO_2_ group and C-N stretching. These changes were explained considering the NIF photodegradation reaction, which leads to the generation of the compound 4-(2-nitrosophenyl)-2.6-dimethyl-3.5-dimethoxy carbonyl pyridine. The interaction of NIF with NaOH in the absence of UV light was demonstrated to induce changes in the vibrational mode of the -C-C-O bond in the ester group. The photodegradation of NIF after its reaction with NaOH induces significant changes highlighted in its (a) UV-VIS spectra, by the shift of the absorption band at 238 nm; (b) PL spectra, by the supraunitary value of the ratio between the emission band intensities at 394–396 nm and 450 nm; (c) Raman spectra, by the change in the ratio between the intensities of the lines that peaked at 1224 cm^−1^ and 1649 cm^−1^ from 0.61 to 0.49; and (d) FTIR spectra, by the lowered absorbance of the IR band at 1493 cm^−1^ assigned to the vibrational mode of the -NO_2_ group as a result of the generation of the nitroso compound. These changes were explained considering the hydrolysis reaction products of NIF, as the nitroso compound is converted to a lactam-type compound. The photodegradation reaction rate constants of NIF and NIF after interaction with NaOH were also reported. The decrease in thermal stability of NIF samples after interaction with NaOH, as well as of NIF after exposure to UV light compared to NIF prior to exposure to UV light, was demonstrated by thermogravimetry, and the key fragments were confirmed by mass spectrometry.

## 1. Introduction

Nifedipine (NIF) is the active compound of drugs prescribed to regulate hypertension [1], angina pectoris [2], premature labor [3], Raynaud’s phenomenon and Chilblains [4], malignant hyperthermia [5], heart disorders [6] and chronic kidney disease [7]. Early studies have shown that NIF can also be used to monitor the metabolism of chondrocytes, as well as stem cells produced from bone marrow [8]. Great effort was made to improve the bioavailability and retention of this drug in the gastrointestinal tract when new drug delivery systems were developed [9]. Studies concerning the photodegradation of NIF began in 1992 [10]. The photodegradation products of NIF in hospital prescriptions were reported in 1994 [11]. According to Refs. [12,13], the therapeutic effect of drugs containing NIF was diminished due to photodegradation processes and toxic products. Various methods to assess the photostability of NIF, such as Fourier-transform reflection–absorption infrared spectroscopy [14], liquid chromatography [15], ^1^H–^14^N NMR–NQR [16], X-ray diffraction [17] and UV-VIS spectroscopy [18], were used. Spectrophotometric evaluation of NIF concentrations in pharmaceutical formulations was performed considering the reaction of NIF with an alkaline solution in dimethyl sulfoxide [19]. This method was reported to be sensitive for determining low NIF concentrations [19]. However, a key factor that can negatively influence the determination of NIF concentrations in pharmaceutical formulations is UV light.

Early studies showed that (i) NIF, i.e., 1, 4-dihydro-2.6-dimethyl-4-(2-nitrophenyl)-3,5-pyridine carboxylate, by exposure to UV light, is transformed into 4-(2-nitrosophenyl)-2.6-dimethyl-3.5-dimethoxy carbonyl pyridine and 4-(2-nitrophenyl)-2.6-dimethyl-3.5-dimethoxy carbonyl pyridine [20]; (ii) under in vitro conditions, NIF is sensitive to UV light, leading to nitroso derivatives which subsequently can react, resulting in compounds of the lactam type, according to high-purity liquid chromatography (HPLC) analysis [21]; (iii) NIF in the presence of hemoglobin at pH > 10 is unstable [22]; and (iv) pH changes alter the sensitivity of vascular tissue to NIF in the case of ischemia, inhibiting contractions induced by the agonist [23]. A removal rate of NIF of 65% was induced by photodegradation processes [24], while in the presence of UV light and H_2_O_2_, the NIF removal rate was increased to 99.94% [25].

Taking into account this progress, in this manuscript, using correlated studies of UV-VIS spectroscopy, Raman scattering, FTIR spectroscopy and mass spectrometry, the influence of UV light on the degradation of NIF interacting with alkaline solutions will be presented. We will demonstrate that in addition to UV-VIS spectroscopy, which is often used to illustrate the photodegradation of drugs, including NIF, alternative methods such as photoluminescence (PL) and/or photoluminescence excitation (PLE) can be used. Potential structural changes that may occur because of the hydrolysis reaction of NIF will be revealed by FTIR spectroscopy and Raman scattering. Additional confirmation of the photodegradation of NIF in the presence of alkaline solutions will be shown by mass spectrometry, and key fragments will be identified.

## 2. Materials and Methods

NIF and anhydrous NaOH pellets, both with purity ≥98%, were purchased from Sigma-Aldrich. The drug commercialized as Nifedipine Retardant was purchased from a local pharmacy. The Nifedipine Retardant drug (NIF-D) contains (a) 20 mg of NIF as the active material; (b) a core consisting of colloidal SiO_2_, microcrystalline cellulose, corn starch, magnesium stearate, lactose monohydrate and polysorbate 80; and (c) a film of TiO_2_ (E171), polyacrylate dispersion 30%, talc, Fe_2_O_3_ (E172), macrogol 6000 and Hypromellose. In these studies, aqueous solutions of NIF (0.6 mM) and NaOH (0.3 M) were prepared. Three aqueous solutions were prepared by mixing (a) 2 mL NIF 0.6 mM with 1 mL NaOH 0.3 M, (b) 1.5 mL NIF 0.6 mM with 1.5 mL NaOH 0.3 M, and (c) 1 mL NIF 0.6 mM with 2 mL NaOH 0.3 M. For the samples using NIF-D, each drug tablet was ground and solubilized in 10 mL distilled water by ultrasonication for 20 min, followed by filtration to separate NIF from the other chemical compounds in the core and film of NIF-D.

UV-VIS spectra of NIF and NIF interacting with NaOH were recorded with a Perkin Elmer Lambda 950 UV-VIS-NIR spectrophotometer. The photodegradation of NIF samples was performed with a mercury-vapor lamp having a power of 350 W. Using a UG5 optical filter, in the 200–380 nm spectral range, the most intense Hg spectral line is at 253 nm. In the case of studies using UV-VIS spectroscopy, the alternation between irradiation times and measurement times was (i) 2 min UV irradiation and 4 min for recording the first three UV-VIS spectra and (ii) 5 min of UV irradiation and 4 min for recording the next twenty UV-VIS spectra. The total time of UV irradiation was 136 min, while the total time of recording the UV-VIS spectra was 544 min.

PL and PLE spectra of NIF and NIF interacting with NaOH were recorded with a Fluorolog-3 spectrophotometer, FL-3.2.1.1 model, from Horiba Jobin Yvon, endowed with a 450 W Xe lamp. The excitation wavelength (λ_exc_) for recording PL spectra was 300 nm, while the emission wavelength (λ_em_) of PLE spectra was equal to 480 nm. The times for the exposure of samples to UV light and for measurement were (a) 136 min and 224 min, respectively, for PL spectra, with an alternation of 60 sec UV light and 302 sec for recording the PL spectrum (without UV range), and (b) 136 min UV light and 90 min, respectively, for PLE spectra, with an alternation of 60 sec UV light and 40 sec for recording the PLE spectrum (without UV range).

Raman spectra of NIF and NIF interacting with NaOH were obtained using a MultiRam FT-Raman spectrophotometer from Bruker, in the backscattering geometry.

FTIR spectra of NIF and NIF interacting with NaOH were recorded with a Vertex 80 FTIR spectrophotometer from Bruker, endowed with an attenuated total reflection (ATR) device.

For the analysis by Raman scattering and FTIR spectroscopy, the samples were in solid state, being obtained by evaporating water from samples: (a) 3 mL NIF 0.6 mM and (b) 1 mL NIF 0.6 mM with 2 mL NaOH 0.3 M. The process took place in a vacuum for 7 days until the two samples had a constant mass.

The thermal analysis measurements were performed with a SETARAM Setsys Evolution 18 Thermogravimeter in the TG-DSC mode. Samples with an initial mass of ≈ 16 mg were heated from 25 °C to 900 °C (sample NIF) and 430 °C (samples of photodegraded NIF and NIF interacting with NaOH) with a heating rate of 5 °C/min. The experiments were conducted in synthetic air (20% O_2_, 80% N_2_) with a gas flow rate of 16 mL/min. The accuracy of the heat flow measurements and the temperature precision were ± 0.001 mW and ±0.01 °C. For the monitoring of gases and molecular fragments obtained from the thermal decomposition, a Setaram QMS 301 Omnistar Pfeiffer mass spectrometer was used. This gas analyzer was coupled to the Setaram Setsys Evolution 18 device. The acquisition of data was performed in multiple ion detection (MID) mode. Gases and molecular fragments were calculated by multiplying the intensities by previously determined calibration factors [26]. The MS data were recorded through calibration of the ambient air species (argon, nitrogen and oxygen). The NIST database library was used for the assignment of fragments [27].

## 3. Results and Discussion

**Figure 1:**
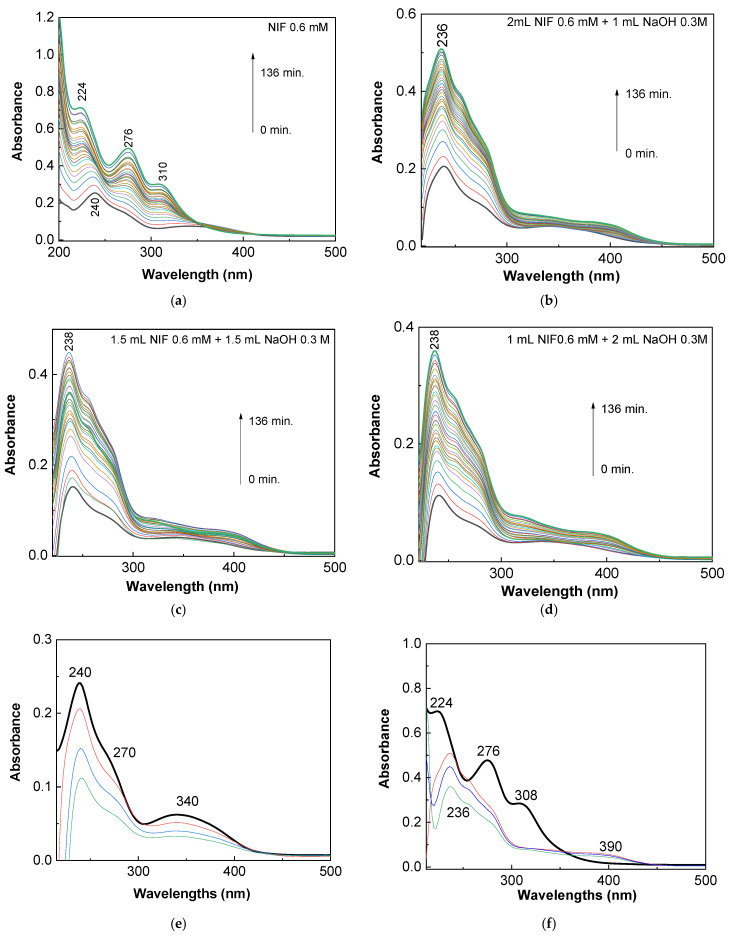
UV-VIS spectra of 0.6 mM NIF solution (**a**) and of mixtures consisting of (**b**) 2 mL NIF 0.6 mM + 1 mL NaOH 0.3M, (**c**) 1.5 mL NIF 0.6 mM + 1.5 mL NaOH 0.3M and (**d**) 1 mL NIF 0.6 mM + 2 mL NaOH 0.3M and their variations during 136 min UV irradiation. (**e**,**f**) show UV-VIS spectra of the samples of NIF (black curves), 2 mL NIF 0.6 mM + 1 mL NaOH 0.3M (red curves), 1.5 mL NIF 0.6 mM + 1.5 mL NaOH 0.3M (green curves) and 1 mL NIF 0.6 mM + 2 mL NaOH 0.3M (blue curves) before and after 136 min of UV irradiation, respectively.

The UV-VIS spectrum of NIF (black curve in Figure 1a) presents a band of high absorbance at 240 nm, with a shoulder at 272 nm, and another band at 340 nm. Bands at 240 nm (5.2 eV), 272 nm (4.6 eV) and 340 nm (3.6 eV) were associated with the electronic transitions of the chromophore groups -COOCH_3_ [28] and -NO_2_ [29] and a heterocycle with six atoms [30]. The ratio between the absorbances of the bands at 240 nm, 270 nm and 340 nm is 3.6:2:1.

**Scheme 1:**
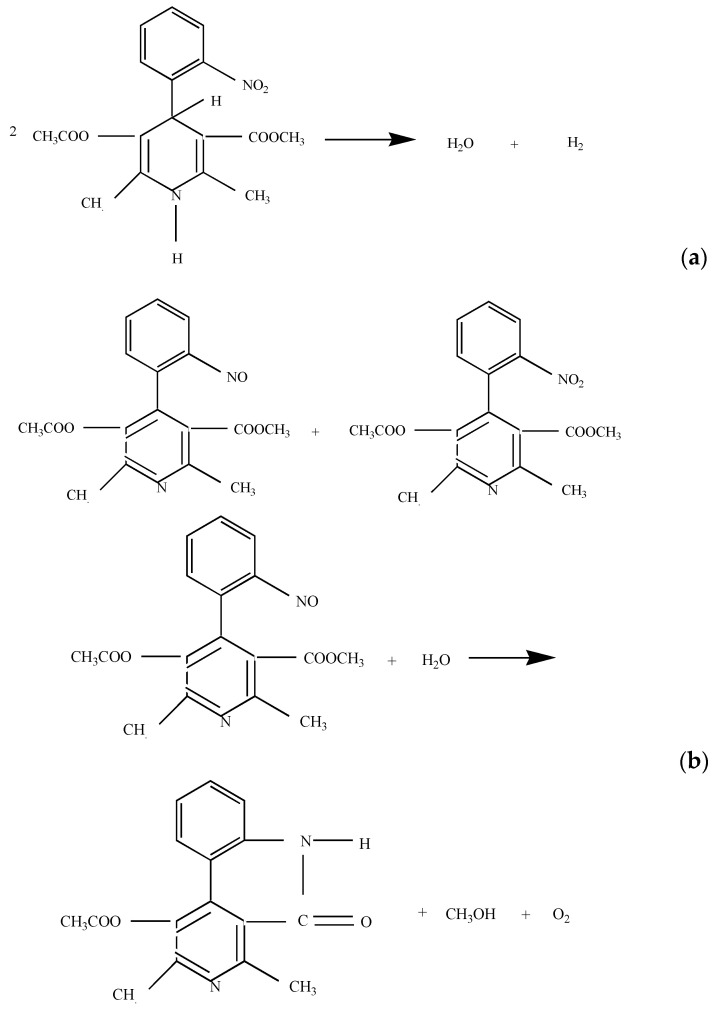
Photodegradation of 1, 4-dihydro-2.6-dimethyl-4-(2-nitrophenyl)-3,5-pyridine carboxylate (NIF) by exposure to UV light, resulting in 4-(2-nitrosophenyl)-2.6-dimethyl-3.5-dimethoxy carbonyl pyridine and 4-(2-nitrophenyl)-2.6-dimethyl-3.5-dimethoxy carbonyl pyridine (**a**). Interaction of 4-(2-nitrosophenyl)-2.6-dimethyl-3.5-dimethoxy carbonyl pyridine with water in the presence of UV light (**b**).

The exposure of NIF to UV light induces a high-energy shift of the bands from 240 nm and 340 nm to 224 nm and 310 nm, respectively. After 136 min UV irradiation, the ratio between the absorbances of bands at 224 nm, 276 nm and 310 nm is equal to 2.4:1.6:1. Recently, a band at 310 nm was reported in the case of NIF in methanol, which was exposure to UVA light for 2 h [31]. According to Ref. [32], the band peaking at 310 nm is characteristic of nitroso compounds. The decrease in the absorbance of the band attributed to the electronic transition of the -NO_2_ group has its origin in the photo-transformation of NIF in 4-(2-nitrosophenyl)-2.6-dimethyl-3.5-dimethoxy carbonyl pyridine [20,31]. These variations indicate changes in the electronic transitions of NIF, in good agreement with Refs. [31,32]. An explanation for the decrease in the ratio between absorbances of the bands peaking 240 nm and 270 nm, attributed to the electronic transitions of the -COOCH_3_ and -NO_2_ groups, from 3.6:2 to 2.4:1.6 must take into account the instability of 4-(2-nitrosophenyl)-2,6-dimethyl-3,5-dimethoxy carbonyl pyridine which in the presence of water leads to the formation of a lactam-like compound and methanol, according to Scheme 1.

Figure 1e illustrates that the interaction of NIF with NaOH leads to a gradual decrease in the absorbance of the bands with maxima at 240 nm and 340 nm. In the presence of UV light, both in the case of NIF (Figure 1a) and in the case of NIF that interacted with NaOH (Figure 1b–d), an increase in the absorbance of the UV-VIS absorption spectra is observed. According to Figure 1f, at the end of the 136 min of UV irradiation, it is observed that the UV-VIS spectra differ in the case of NIF and the samples of NIF that interacted with NaOH. Increasing the UV irradiation time over 136 min did not lead to other variations in the UV-VIS spectra.

**Figure 2:**
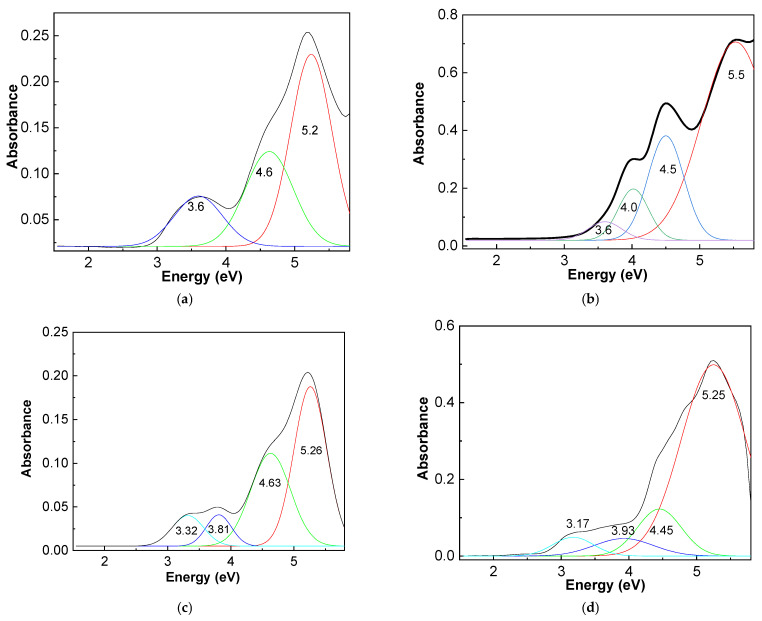

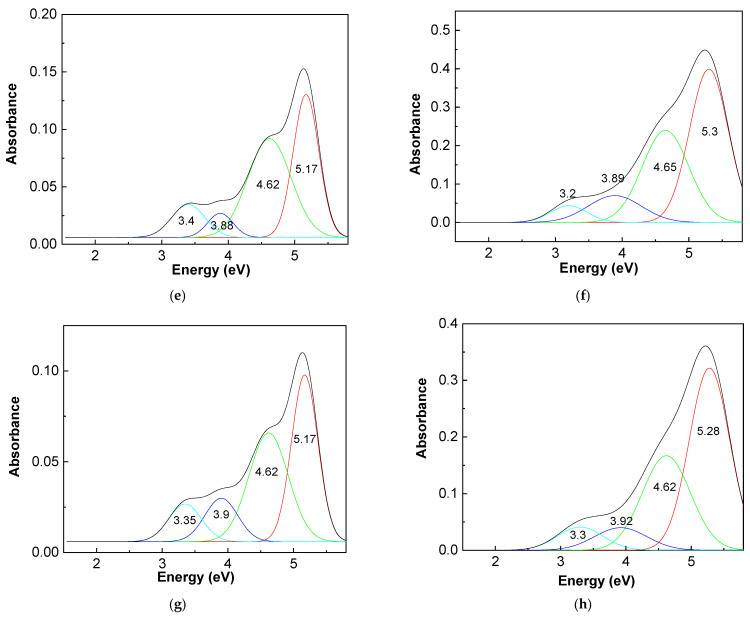
Deconvolution of the UV-VIS spectra of the following samples in initial state and after 136 min UV irradiation: (i) 0.6M NIF (**a**,**b**), (ii) 2 mL NIF 0.6 mM + 1 mL NaOH 0.3M (**c**,**d**), (iii) 1.5 mL NIF 0.6 mM + 1.5 mL NaOH 0.3M (**e**,**f**) and (iv) 1 mL NIF 0.6 mM + 2 mL NaOH 0.3M (**g**,**h**). Black curves correspond to experimental UV-VIS spectra, while cyan, blue, green and red curves correspond to the components resulted by deconvolution of the experimental UV-VIS spectra.

A more detailed analysis of the initial state and the end of the 136 min of UV irradiation of the NIF samples and the samples of NIF that interacted with NaOH is presented in Figure 2. Figure 2 shows the deconvolution of the initial state and the end of the 136 min UV irradiation of the four samples. Figure 2a_,_b highlights that the exposure of NIF to UV light induces a decrease in absorbance of the band at 3.6 eV, simultaneous with the appearance of the band at 4 eV, as a consequence of the generation of the photodegradation product 4-(2-nitrosophenyl)-2,6-dimethyl-3.5-dimethoxy carbonyl pyridine [32].

**Scheme 2:**
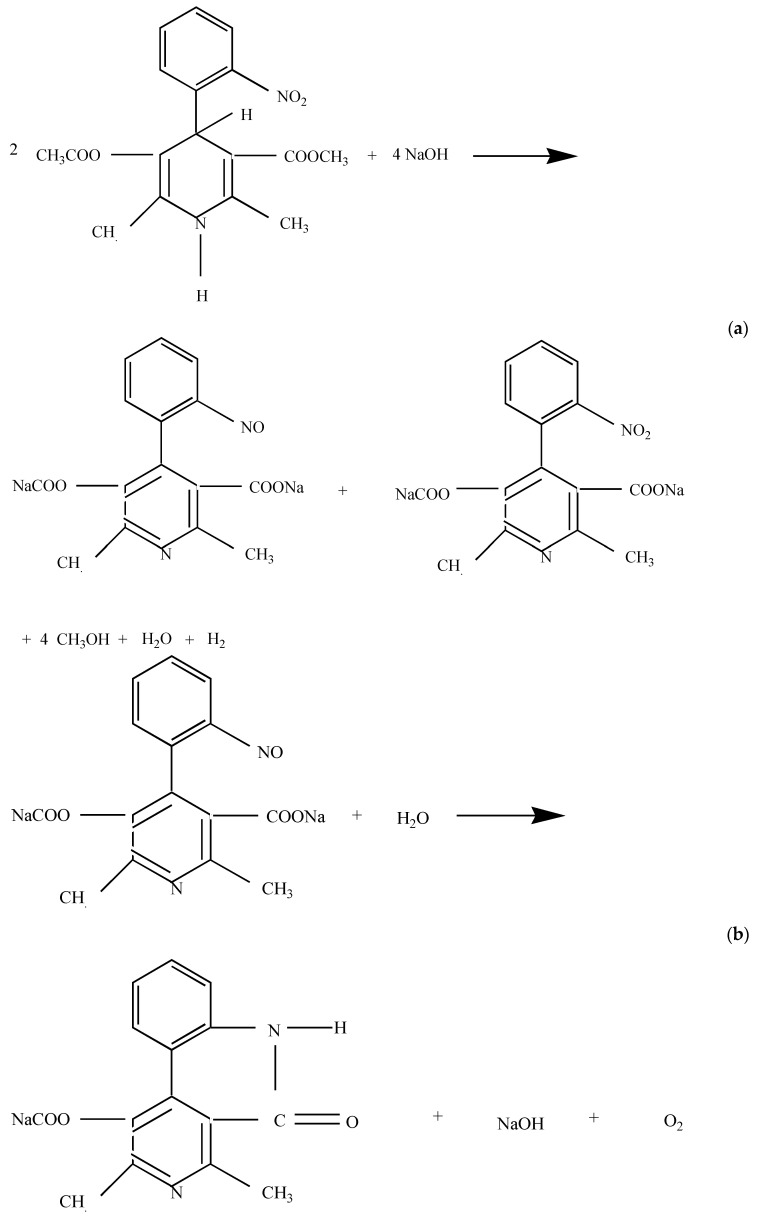
The interaction of NIF with NaOH (**a**) and photo-transformation of the nitroso compound into a lactam-type compound (**b**).

The interaction of NIF with NaOH, before UV irradiation, induces the following changes: (i) a hypsochromic shift of the band from 5.2 eV (Figure 2a) to 5.17eV (Figure 2e,g); (ii) a bathochromic shift of the band from 4.6 eV (Figure 2a) to 4.62–4.63 eV (Figure 2c,e,g); and (iii) a decrease in absorbance of the band localized in the spectral range 2.6–4.25 eV, with the maxima at 3.32–3.35 eV and 3.81–3.9 eV (Figure 2c,e,g). The exposure of NIF after its interaction with NaOH to UV light induces the following in the case of the three samples: (i) a hypsochromic shift of the bands from 3.32 eV to 3.17 eV (Figure 2c,d), 3.4 eV to 3.2 eV (Figure 2e,f), and 3.35 eV to 3.3 eV (Figure 2g,h) and (ii) a bathochromic shift of the bands from 3.81 eV to 3.93 eV (Figure 2c,d), 3.88 eV to 3.89 eV (Figure 2e,f), and 3.9 eV to 3.92 eV (Figure 2g,h). These variations suggest that the interaction of NIF with NaOH leads to the generation of a nitroso compound which, being unstable, is transformed into a lactam-type compound, according to Scheme 2.

Equation (1) is used to calculate the photodegradation reaction rate constant of NIF:

ln (A_0_/A_t_) = k × t (1)

**Figure 3:**
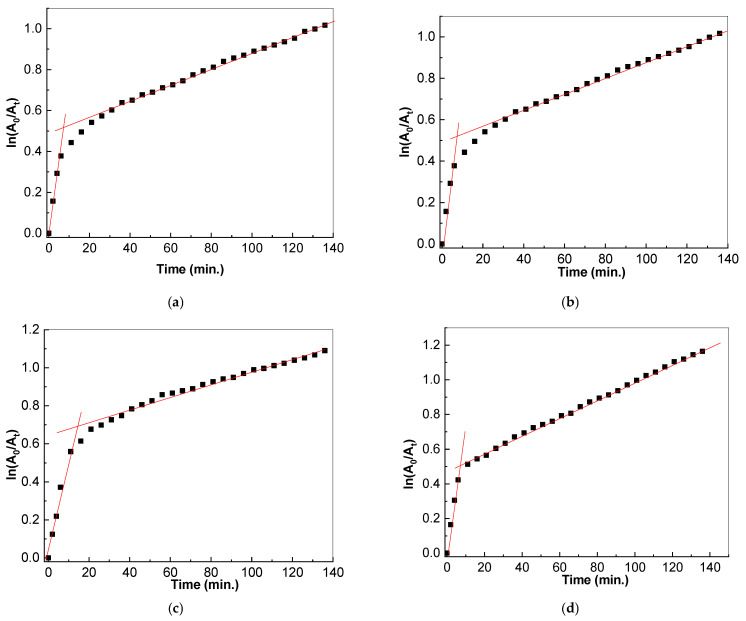
The photodegradation reaction kinetics of the aqueous solutions of (**a**) NIF, (**b**) 2 mL NIF 0.6 mM + 1 mL NaOH 0.3 M, (**c**) 1.5 mL NIF 0.6 mM + 1.5 mL NaOH 0.3 M and (**d**) 1 mL NIF 0.6 mM + 2 mL NaOH 0.3 M. The square dots correspond to the values of ln(A0/At) at different times, while red lines correspond to the linear fit.

A_t_ and A_0_ are the absorbance values of the sample at t = 2–136 min and t = 0 min, while k corresponds to the photodegradation rate constant. Figure 3 highlights two linear regions for all samples. Table 1 shows the reaction rate constant value in the case of the two linear regions, labeled k_1_ and k_2_, and the linear regression coefficients, labeled R_1_^2^ and R_2_^2^, for each sample.

According to Table 1, the values of the rate constants of the photodegradation reactions of NIF that interacted with NaOH are superior to those reported in the case of NIF. The presence of the two linear regions can be explained by considering reaction (**a**) in Scheme 1 and Scheme 2, characterized by k_1_, which leads to the intermediate products of the 4-(2-nitrosophenyl)-2.6-dimethyl-3.5-dimethoxy carbonyl pyridine and 4-(2-nitrophenyl)-2.6-dimethyl-3.5-dimethoxy carbonyl pyridine type or their sodium salts, and reaction (**b**) in Scheme 1 and Scheme 2, in which 4-(2-nitrosophenyl)-2.6-dimethyl-3.5-dimethoxy carbonyl pyridine is transformed into a lactam-type compound.

**Figure 4:**
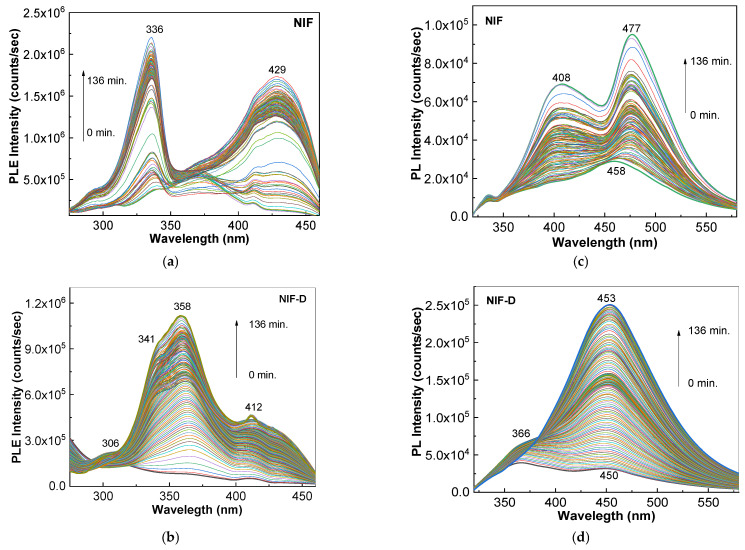
PLE (**a**,**b**) and PL (**c**,**d**) spectra of NIF and NIF-D and their variations during 136 min UV irradiation. PLE spectra were recorded at λ_em_ = 480 nm, while PL spectra were recorded at λ_exc_ = 300 nm.

Figure 4a,c show the PLE and PL spectra of NIF and NIF-D. The PLE spectrum of NIF in the initial state shows three bands at 338 nm, 371 nm and 411 nm, while the PL spectrum shows an emission band with a maximum at 458 nm, which is asymmetric in the high-energy range as a consequence of emission band at 404 nm. The exposure of NIF to UV light induces (i) an increase in the intensities of the PLE spectrum bands from 338 nm and 411 nm, simultaneous with a red-shift of the band from 411 nm to 429 nm, and (ii) a red-shift of the PL spectrum band from 458 nm to 477 nm and an increase in the intensities of the PL bands at 404–408 nm and 458–477 nm. An increase in the intensities of PLE and PL spectra is also reported in the case of NIF-D. Figure 4b,d highlight changes in the ratio between (i) the intensities of the PLE bands with maxima at 358 nm and 410 nm from 1.63, before UV irradiation of NIF-D, to 2.4 after 136 min of UV irradiation of the NIF-D sample and (ii) the intensities of the PL bands situated at 366 nm and 450 nm from 1.25 before UV irradiation of NIF-D to 0.19 after 136 min of UV irradiation of the NIF-D sample.

**Figure 5:**
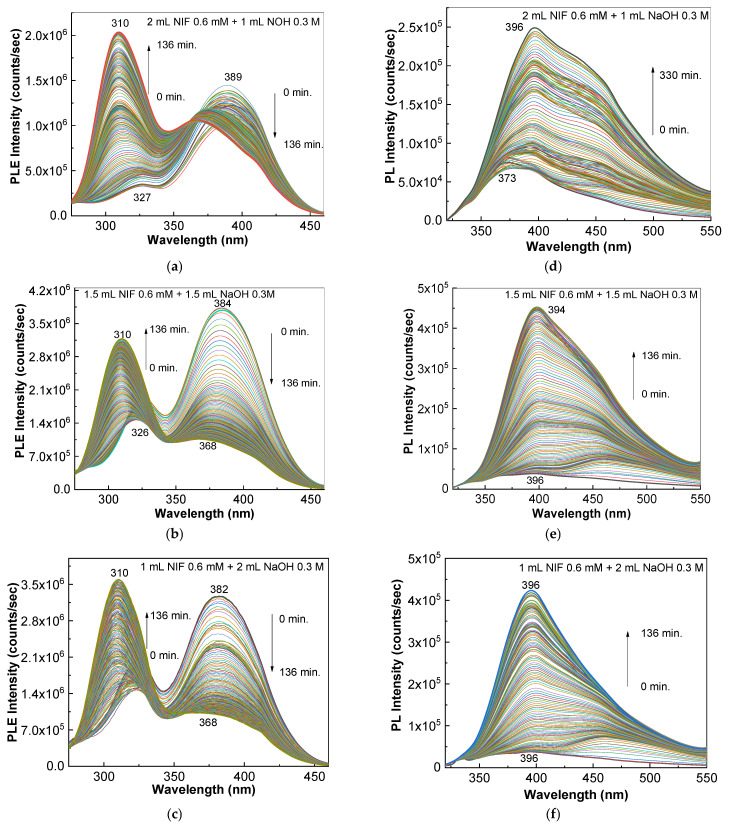
PLE (**a**–**c**) and PL (**d**–**f**) spectra of samples consisting of (i) 2 mL NIF 0.6 mM + 1 mL NaOH 0.3 M (**a**,**d**), (ii) 1.5 mL NIF 0.6 mM + 1.5 mL NaOH 0.3 M (**b**,**e**) and (iii) 1 mL NIF 0.6 mM + 2 mL NaOH 0.3 M (**c**,**f**) and their variations during 136 min UV irradiation. PLE spectra were recorded at λ_em_ = 480 nm, while PL spectra were recorded at λ_exc_ = 300 nm.

The PLE and PL spectra of the NIF samples that interacted with NaOH are shown in Figure 5. Before UV irradiation, in all cases shown in Figure 5a–c, a PLE band of low intensity at 325–327 nm and another one of high intensity at 382–389 nm are observed. The ratio of the PLE band intensities at 325–327 nm and 382–389 nm varies from 0.32 (Figure 5a) to 0.47 (Figure 5b) and 0.63 (Figure 5c). According to Figure 5, after 136 min UV irradiation of samples of NIF that interacted with NaOH, a red-shift of the PLE bands from 325–327 nm and 382–389 nm to 310 nm and 368–370 nm, respectively, is reported.

The ratio of the intensities of PLE bands with maxima at 310 nm and 368–370 nm varies from 1.94 (Figure 5a) to 2.99 (Figure 5b) and 3.47 (Figure 5c). Regarding the PL spectra of the samples of NIF after interaction with NaOH, before UV irradiation (Figure 5d–f), they show a different profile in contrast with the PL spectrum of NIF (Figure 4c). Thus, we observe that for the three samples of NIF after interaction with NaOH, the most intense PL band peaks at 373 nm (Figure 5d) and 396 nm (Figure 5e,f), showing an asymmetric profile in the range of low energies because of the PL band at 450 nm. Before exposure to UV light, the ratio between the intensities of the PL bands of NIF at 408 nm and 458 nm is equal to 0.65 (Figure 4c), while for the three samples of NIF after interaction with NaOH, the ratio between the intensities of PL bands at 373–396 and 450 nm has values greater than 1, i.e., 2.8 (Figure 5d), 1.5 (Figure 5e) and 1.7 (Figure 5f). After 136 min UV irradiation, we find the same behavior; the ratio of PL band intensities at 394–396 nm and 450 nm is supraunitary, i.e., 1.4 (Figure 5d), 1.63 (Figure 5e) and 2.23 (Figure 5f), values which are higher than that of NIF (0.73, Figure 4c).

**Figure 6:**
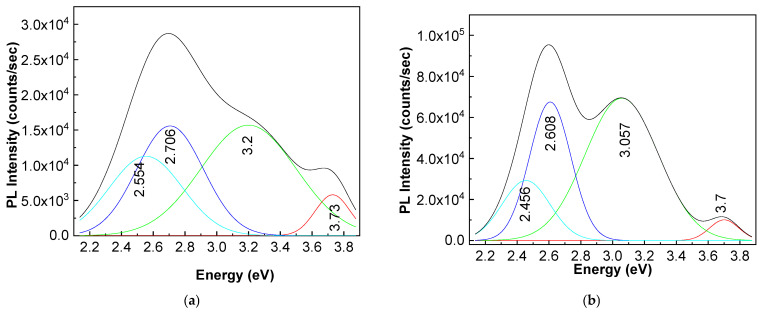

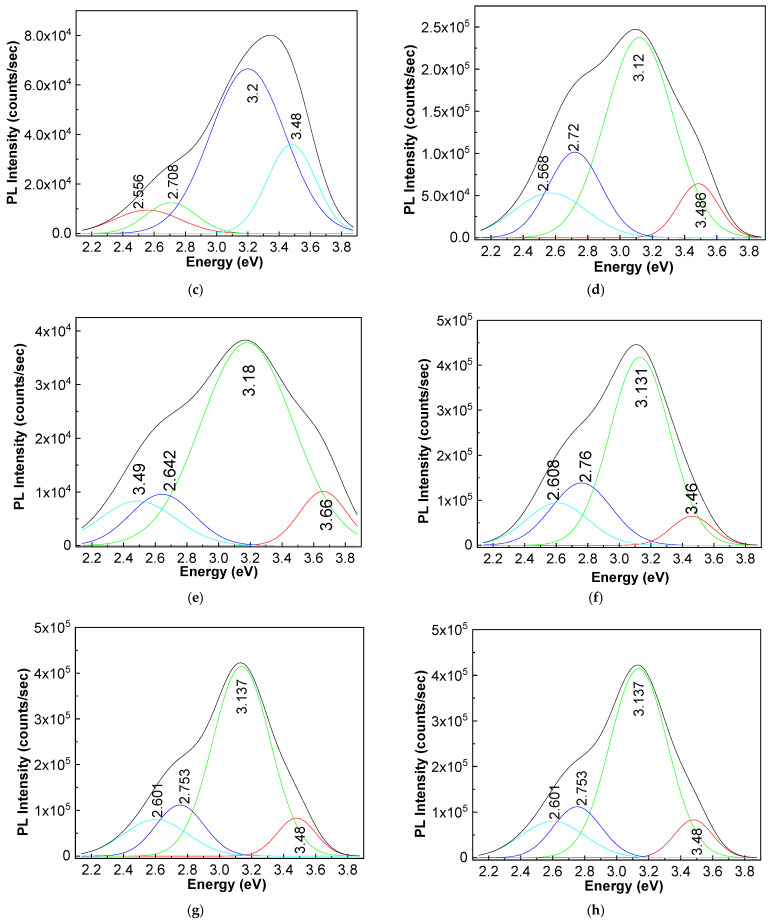
Deconvolution of PL spectra of samples of the types NIF (**a**,**b**), 2 mL NIF 0.6 mM + 1 mL NaOH 0.3 M (**c**,**d**), 1.5 mL NIF 0.6 mM + 1.5 mL NaOH 0.3 M (**e**,**f**) and 1 mL NIF 0.6 mM + 2 mL NaOH 0.3 M (**g**,**h**), before and at the end of 136 min UV light irradiation. Black curves correspond to experimental PL spectra, while cyan, blue, green and red curves correspond to the components resulted by deconvolution of the experimental PL spectra.

Figure 6 shows the deconvolution of PL spectra of NIF and samples of NIF that interacted with NaOH, before and at the end of 136 min UV irradiation. The deconvolution of the PL spectra of all samples, before and after 136 min UV irradiation, reveals four emission bands. Thus, in the case of NIF, the four PL bands peak at 2.554 eV (A band), 2.706 eV (B band), 3.2 eV (C band) and 3.73 eV (D band). The interaction of NIF with NaOH induces a change in the profile of PL spectra as well as in the ratio between the intensity of the four PL bands, i.e., A, B, C and D, which is (a) 0.37:1:1:0.72 (Figure 6a), (b) 2.89:5.33:1:0.75 (Figure 6c), (c) 1.05:3.96:1:0.87 (Figure 6e) and (d) 1.67:4.44:1:1.08 (Figure 6g). After 136 min UV irradiation, both in the case of NIF and in the case of samples of NIF that interacted with NaOH, changes are induced in the ratio between the intensities of the four PL bands, which is (a) 0.15:1.03:1:0.43 (Figure 6b), (b) 0.63:2.34:1:0.52 (Figure 6d), (c) 0.46:3.01:1:0.67 (Figure 6f) and (d) 0.74:3.7:1:0.71 (Figure 6h), along with a down-shift of the four emission bands.

The most important variations in emission bands in Figure 6 are those in the bands labeled as A and B; the frequency separation between these two emission bands is 0.152 eV, which corresponds to the wavenumber 1226 cm^−1^, a value that is close to the Raman line of NIF situated at 1224 cm^−1^ which was assigned to the vibrational mode of the C-C-O bond in the ester group [23]. We anticipate that the studies employing Raman scattering will illustrate the changes related to the C-C-O bond in the ester group of NIF.

**Figure 7:**
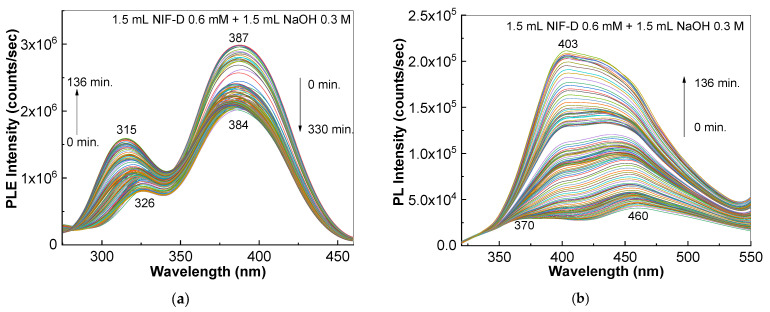
PLE (**a**) and PL (**b**) spectra of 1.5 mL NIF-D containing 0.6 mM nifedipine after interaction with 1.5 mL NaOH 0.3 M under UV irradiation for 136 min. PLE spectra were recorded at λ_em_ = 480 nm, while PL spectra were recorded at λ_exc_ = 300 nm.

Figure 7 reveals the variations in the PLE and PL spectra of the sample of NIF-D after interaction with NaOH.

According to Figure 7, the following observations can be made: (i) The PLE spectrum of the NIF-D sample that interacted with NaOH before UV irradiation shows two bands at 326 nm and 387 nm, and the ratio between their intensities is 0.35 (Figure 7a), a lower value than that reported for NIF-D, i.e., 1.63 (Figure 4b); after 136 min UV light exposure, a red-shift of the PLE band from 315 nm to 321 nm (Figure 7a) and a change in the ratio of the PLE band intensities from 321 nm and 384 nm at 0.72 take place. This value is smaller than reported for NIF-D, i.e., 2.4 (Figure 4b). (ii) The PL spectrum of the NIF-D sample after interaction with NaOH before UV irradiation shows two bands at 370 nm and 460 nm (Figure 7b), and the ratio between their intensities is 0.75, a value which is lower than that of NIF-D, i.e., 1.25 (Figure 4d); after 136 min UV light exposure, a red-shift of the PL band from 370 nm to 403 nm occurs, and the ratio between the intensities of the PL bands at 403 nm and 450 nm is 1.15, which is less than that reported for NIF-D, i.e., 0.19 (Figure 4d).

An explanation for the changes reported in the PLE and PL spectra of NIF and NIF-D after interaction with NaOH must consider the reactions shown in Scheme 2.

**Figure 8:**
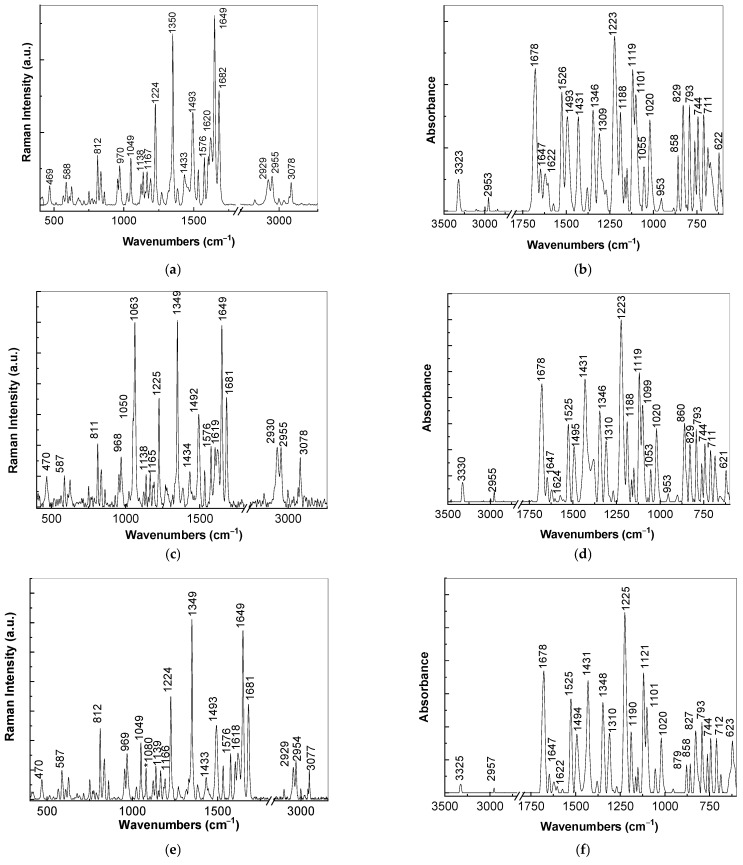

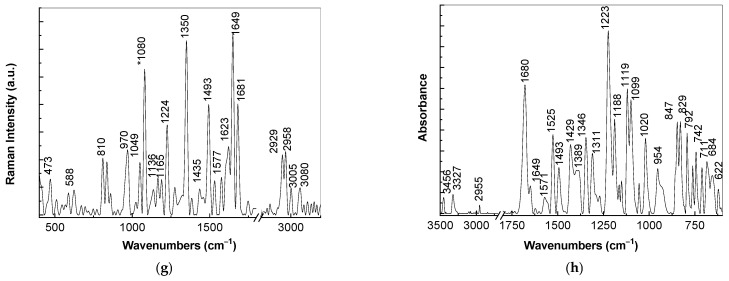
The Raman and FTIR spectra of NIF in its initial state (**a**,**b**) and after its photodegradation (**c**,**d**) as well as NIF that interacted with NaOH before (**e**,**f**) and after its photodegradation (**g**,**h**).

To support this statement, the Raman and FTIR spectra of NIF and NIF that interacted with NaOH before and after 136 min of UV irradiation are presented. According to Figure 8a, the Raman lines of NIF are situated at 469–588 cm^−1^, 812 cm^−1^, 970 cm^−1^, 1049 cm^−1^, 1224 cm^−1^, 1350 cm^−1^, 1493 cm^−1^, 1576 cm^−1^, 1620 cm^−1^, 1649 cm^−1^, 1682 cm^−1^, 2929–2955 cm^−1^ and 3078 cm^−1^, and their assignment is shown in Table 2.

Figure 8b highlights the IR bands of NIF at 622–712, 744–792, 829–858, 953, 1020–1188, 1223, 1309–1431, 1493, 1526, 1622, 1647–1678, 2953 and 3323 cm^−1^, and their assignment is presented in Table 3.

After the exposure of NIF to UV light, the appearance of a new Raman line at 1063 cm^−1^ simultaneously with the increase in the intensity of the Raman line peaking at 1050 cm^−1^ is noted (Figure 8c). The Raman line at 1063 cm^−1^ belongs to the lactam-ring breathing [36]. The presence of an IR band of low absorbance at 903 cm^−1^ is observed in Figure 8d and is assigned to the asymmetric stretching of C-C bonds in a lactam-type compound [37]. According to Figure 8e, the interaction of NIF with NaOH leads to the appearance of a new Raman line at 1080 cm^−1^ which belongs to the alkoxide group vibrational mode [38]. The UV exposure of NIF that reacted with NaOH leads to (a) a variation in the ratio between the intensities of the Raman lines peaking at 1224 cm^−1^ and 1649 cm^−1^, associated with the vibrational modes of -C-C-O in the ester group and C=C stretching (I_1224_:I_1649_), from 0.61:1 (Figure 8e) to 0.49:1 (Figure 8g) and (b) the appearance of new Raman line at 3005 cm^−1^. Other changes induced by the exposure of NIF to UV light are as follows: (i) a change in the ratio between the absorbances of the IR bands peaking at 1020 cm^−1^ and 1223 cm^−1^, associated with the vibrational modes of -C-O-C- and C-N stretching (A_1020_:A_1223_), from 0.52:1 (Figure 8b) to 0.4:1 (Figure 8d); (ii) a shift of the IR band from 3323 cm^−1^ to 3330 cm^−1^; and (iii) a decrease in the ratio between the absorbances of the IR bands peaking at 1493 cm^−1^ and 1223 cm^−1^, associated with the vibrational modes of the -NO_2_ group and C-N stretching (A_1493_:A_1223_), from 0.54:1 (Figure 8b) to 0.3:1 (Figure 8d). The lower values of the ratios A_1020_:A_1223_ and A_1493_:A_1223_ indicate a lower share of the ester and nitro groups after the exposure of NIF to UV light.

The interaction of NIF with NaOH before exposure to UV light induces a variation in the ratio A_1493_:A_1223_ from 0.54 (Figure 8b) to 0.32 (Figure 8f), a fact which indicates a decrease in the nitro groups’ weight in the reaction product mass. After that NIF interacted with NaOH and was exposed to UV light, the ratio A_1493_:A_1223_ was equal to 0.25 (Figure 8h), a value which indicates that a diminution in the nitro groups’ weight takes place. In addition, the appearance of a new IR band peaking at 3456 cm^−1^ is observed in Figure 8h. Early studies on the IR active vibrational modes of methanol clearly demonstrated that a band at 3456 cm^−1^ was attributed to the OH stretching vibrations of methanol [39]. Figure 8h reveals the appearance of a new complex band with a maximum at 954 cm^−1^ and a shoulder at 931 cm^−1^, and this band was assigned to NC stretching in a lactam-type compound [37]. The lower intensity of the Raman line at 1224 cm^−1^ (in Figure 8g compared to Figure 8e), which was attributed to the vibrational mode of the -C-C-O bonds in the ester group, and the lower absorbance of the IR band at 1020 cm^−1^ (in Figure 8d in contrast with Figure 8b) assigned to the vibrational mode of the -C-O-C bond indicate a decrease in the weight of ester groups. An explanation for the lower intensity of the Raman line at 1224 cm^−1^ and lower absorbance of the IR bands at 1020 cm^−1^ and 1493 cm^−1^ must take into account Scheme 1 and Scheme 2. In our opinion, Scheme 1 and Scheme 2 explain (i) the diminution in the weight of ester groups, as a result of the transformation of -COOCH_3_ groups into -COOH groups, and (ii) the decrease in the share of -NO_2_ groups as a result of the generation of compounds containing -NO groups.

**Figure 9:**
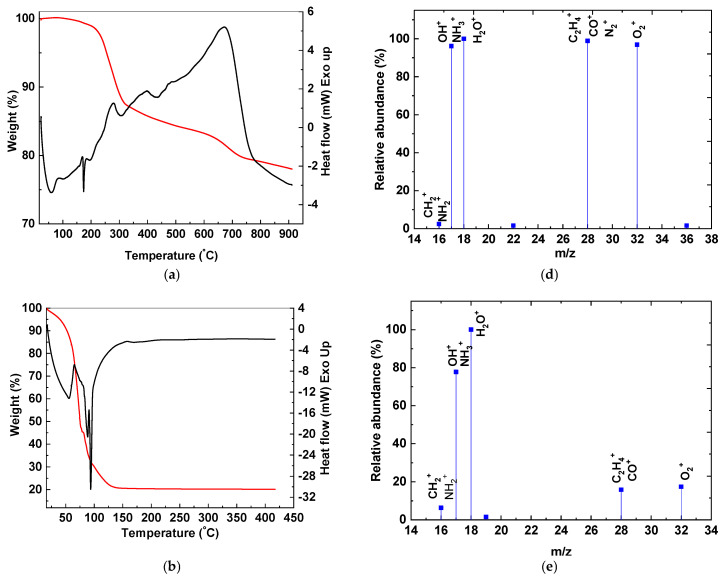

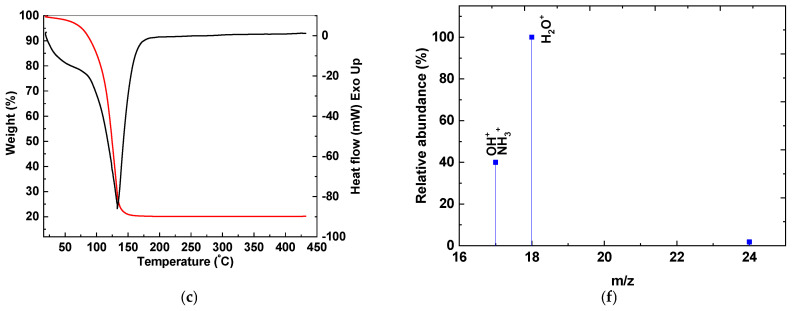
TG-DSC curves of NIF in its initial state (**a**) and after photodegradation for 136 min in the absence (**b**) and presence of NaOH (**c**). (**d**–**f**) show the MS curves of NIF in its initial state, NIF after 136 min of UV irradiation and NIF that interacted with NaOH and was then subjected to UV irradiation for 136 min, respectively. Black and red curves correspond to DSC and TG curves, respectively.

An additional confirmation of Scheme 1 and Scheme 2 is shown by the analysis of TG-DSC and MS (Figure 9). The TG-DSC curves corresponding to NIF before exposure to UV light (Figure 9a) reveal a thermal degradation of NIF that occurs in three stages. The first sharp, endothermic peak highlighted on the DSC curve, at 174 °C, represents the melting point of NIF. This is accompanied by a succession of peaks, two endothermal peaks with maximums at 305 °C and 433 °C and a wide, exothermic peak centered at 674 °C corresponding to a slow process of thermal decomposition of NIF with the continuous formation of metastable phases that decompose as the temperature rises. The total mass loss recorded on the TG curve is about 22%. These data are like those reported for this type of material [40,41]. The TG-DSC curves shown in Figure 9b,c corresponding to the samples of NIF and NIF that interacted with NaOH, recorded after UV irradiation, reveal a significant decrease in the thermal stability of these compounds. Thus, after UV irradiation of NIF, the TG curve records a thermal decomposition of NIF in a single stage accompanied by a mass loss of about 80% (Figure 9b). This thermal degradation is evidenced on the corresponding DSC curve of a succession of endothermal peaks with maximums located at 55, 88 and 95 °C, much lower compared to those of the initial sample.

These peaks are most likely due to the formation of metastable phases that are decomposed in a very short period. The sample containing NaOH records a mass loss like that of the sample that was subjected to UV irradiation, about 80% (Figure 9c). This process is accompanied by the presence of a single, high-intensity endothermic peak (DSC curve) with a maximum at 133 °C. This sample has the lowest thermal stability, its decomposition being achieved in a single stage, without the formation of intermediate phases. Although thermal analysis is a method used to identify the thermal decomposition mechanisms of a substance, in the case of drugs, these mechanisms are complex and difficult to identify [42,43]. The different percentages of mass loss (20% for the initial sample versus about 80% for the UV-irradiated sample in the presence of NaOH) are due to the influence of the two factors on the thermal stability of NIF. This represents an element of originality that has not been highlighted so far, justifying the use of thermal analysis as a method to characterize this material. It should also be mentioned that an analysis of the compounds resulting from the thermal decomposition was not performed, as this study focused only on the investigation of factors that could modify the stability of the material. For the first time, the gases resulting from the thermal decomposition of NIF were qualitatively quantified by MS (Figure 9d). MS has begun to be used more and more often in the development of the pharmaceutical industry as an analytical method for evaluating material stability [44,45]. This method has a significant role both in refining drug synthesis and in identifying possible impurities and/or material defects [46]. The analysis of the three mass spectra (Figure 9d–f) confirms the influence of external factors on the material’s thermal stability, both by the recorded number of molecular fragments and by their relative abundance. Due to the complex thermal decomposition mechanisms, the analysis of the resulting gases is difficult to explain, but it shows the instability of the initial substance in response to external factors.

## 4. Conclusions

This work reports new results regarding the photodegradation of NIF interacting with alkaline aqueous solutions reported by UV-VIS spectroscopy, PL, FTIR spectroscopy, Raman scattering, thermogravimetry and mass spectrometry. The results reported in this work lead to the following conclusions:

(a) The photodegradation of an aqueous solution of NIF in the presence of UV light leads to the generation of the compound 4-(2-nitrosophenyl)-2.6-dimethyl-3.5-dimethoxy carbonyl pyridine; this transformation was highlighted by the decrease in the ratio between the intensities of the Raman lines peaking at 1224 cm^−1^ and 1649 cm^−1^, associated with the vibrational modes of -C-C-O in the ester group and C=C stretching, and the decrease in the ratio between the absorbances of the IR bands peaking at 1493 cm^−1^ and 1223 cm^−1^, associated with the vibrational modes of the -NO_2_ group and C-N stretching.
(b) The UV-VIS spectrum of NIF shows three bands at 240 nm, 272 nm and 340 nm which are associated with the electronic transitions of the chromophore groups -COOCH_3_ and -NO_2_ and a heterocycle with six atoms; the exposure of NIF to UV light induces a high-energy shift of the bands from 240 nm and 340 nm to 224 nm and 310 nm, respectively, due to the generation of the nitroso compound. According to studies using UV-VIS spectroscopy, the reaction rate constants of the two linear regions were attributed to (i) the generation of intermediate products of the 4-(2-nitrosophenyl)-2.6-dimethyl-3.5-dimethoxy carbonyl pyridine and 4-(2-nitrophenyl)-2.6-dimethyl-3.5-dimethoxy carbonyl pyridine type or their sodium salts and (ii) the transformation of 4-(2-nitrosophenyl)-2.6-dimethyl-3.5-dimethoxy carbonyl pyridine into a compound containing a lactam ring, and these processes were characterized by k_1 =_ 0.063 min^−1^ and k_2_ = 0.0035 min^−1^.
(c) After 136 min UV irradiation of a sample of NIF that interacted with NaOH, a hypsochromic shift of the absorption band from 3.32–3.4 eV to 3.17–3.3 eV accompanied by a decrease in the absorbance of the 4.62–4.63 eV band was reported; the shift of the absorption band at 5.2 eV indicated that the photodegradation of NIF that interacted with NaOH induces changes in the electronic transition of the -COOCH_3_ groups.
(d) The PL spectra of NIF and the sample of NIF that interacted with NaOH, before and after the photodegradation process, highlight four emission bands in the spectral range 2.1–3.9 eV, where the frequency separation of the two emission bands from the low-energy range is equal to 0.152 eV, a value that corresponds to the Raman line of NIF situated at 1224 cm^−1^ assigned to the vibrational mode of the C-C-O bond in the ester group. The reported changes in PL spectra during the exposure of the NIF samples to UV light indicate that this method is an alternative method to UV-VIS spectroscopy to illustrate the drug’s photodegradation processes.
(e) The change in the ratio between the intensities of Raman lines peaking at 1224 cm^−1^ and 1649 cm^−1^, from 0.61 to 0.49, and the lower absorbance of the IR bands at 1020 cm^−1^ and 1493 cm^−1^ are consequences of the decrease in the weight of ester groups, as a result of the transformation of -COOCH_3_ groups into -COOH groups, and the decrease in the weight of -NO_2_ groups as a result of the generation of compounds containing -NO groups.
(f) The lactam-type compound resulting from the exposure of NIF to UV light was highlighted by the Raman line peaking at 1063 cm^−1^ and the IR band at 903 cm^−1^; after the NIF sample that interacted with NaOH was subjected to UV irradiation, the presence of the lactam-type compound was evidenced by the IR band at 954–931 cm^−1^.
(g) A decrease in the thermal stability of NIF samples after interaction with NaOH and after exposure to UV light compared to the thermal stability of NIF before exposure to UV light was demonstrated by thermogravimetry, and key fragments were confirmed by mass spectrometry.

## Figures and Tables

**Table 1:** Reaction rate constants and linear regression coefficients of the samples of NIF before and after its interaction with NaOH.

Sample	$k_{1}$(min^−1^)	$R_{1}^{2}$	$k_{2}$(min^−1^)	$R_{2}^{2}$
3 mL NIF	0.063	0.9835	0.0035	0.9986
2 mL NIF 0.6 mM + 1 mL NaOH 0.3M	0.078	0.9947	0.0037	0.978
1.5 mL NIF 0.6 mM + 1.5 mL NaOH 0.3M	0.067	0.9968	0.0053	0.994
1 mL NIF 0.6 mM + 2 mL NaOH 0.3M	0.071	0.9946	0.0051	0.9829

**Table 2:** Vibrational modes of NIF observed by Raman spectroscopy.

Wavenumbers (cm^−1^) in This Work	Assignment of Vibrational Modes [33]
469–588	deformation of aromatic ring
812	ring C-H out-of-plane
970	dihydropyridine ring
1049	1, 2-substituted ring
1224	C-C-O in ester
1350	symmetrical stretching NO_2_
1493	C-C in aromatic ring
1576	NH out-of-plane scissoring vibration
1620	C=C in aromatic ring
1649	C=C stretching
1682	C=O stretching
2929–2955	CH aliphatic stretching
3078	CH aromatic stretching

**Table 3:** Vibrational modes of NIF observed by FTIR spectroscopy.

Wavenumbers (cm^−1^) in This Work	Assignment of Vibrational Modes [34,35]
622–712	aromatic ring deformation
744–792	C-C-H out-of-plane bending in benzene ring + wagging NO_2_
829–858	CH wagging
953	C-C-H out-of-plane bending in benzene ring
1020–1188	-C-O-C-
1223	C-N stretching
1309–1431	C-C-H in plane bending in benzene ring
1493	-NO_2,_
1526	-NH bond
1622	pyridine ring
1647–1678	-C=O in ester group
2953	aromatic and aliphatic C-H stretching
3323	-NH group

## Data Availability

Data are contained within the article.
